# Sex-specific effects of a parasite evolving in a female-biased host population

**DOI:** 10.1186/1741-7007-10-104

**Published:** 2012-12-18

**Authors:** David Duneau, Pepijn Luijckx, Ludwig F Ruder, Dieter Ebert

**Affiliations:** 1 University of Basel, Zoological Institute, Vesalgasse 1, 4051, Basel, Switzerland; 2 Department of Entomology, 3132 Comstock Hall, Cornell University, Ithaca, NY 14850, USA; 3 University of Toronto, Department of Ecology and Evolutionary Biology, Toronto, ON M5S 3B2, Canada

**Keywords:** Sex-specific adaptation, Daphnia, Pasteuria, local adaptation, gigantism, castration, biased sex-ratio

## Abstract

**Background:**

Males and females differ in many ways and might present different opportunities and challenges to their parasites. In the same way that parasites adapt to the most common host type, they may adapt to the characteristics of the host sex they encounter most often. To explore this hypothesis, we characterized host sex-specific effects of the parasite *Pasteuria ramosa*, a bacterium evolving in naturally, strongly, female-biased populations of its host *Daphnia magna*.

**Results:**

We show that the parasite proliferates more successfully in female hosts than in male hosts, even though males and females are genetically identical. In addition, when exposure occurred when hosts expressed a sexual dimorphism, females were more infected. In both host sexes, the parasite causes a similar reduction in longevity and leads to some level of castration. However, only in females does parasite-induced castration result in the gigantism that increases the carrying capacity for the proliferating parasite.

**Conclusions:**

We show that mature male and female *Daphnia *represent different environments and reveal one parasite-induced symptom (host castration), which leads to increased carrying capacity for parasite proliferation in female but not male hosts. We propose that parasite induced host castration is a property of parasites that evolved as an adaptation to specifically exploit female hosts.

## Background

Males and females of the same species typically differ in many traits, so much so that the most striking differences among individuals of the same species are usually those between sexes. A given species may have differences in gametes, primary and secondary sexual characters, quality and quantity of hormones, but also differences in behavior, somatic structures, immune response and gene expression [1-4]. These differences can make males and females distinct types of hosts, offering different challenges and opportunities to their parasites. Thus, similar to parasite adaptation to the most common host type [5], parasites may adapt specifically to the characteristics of the host sex they encounter most often [6]. Biased sex-ratios are common and can be an intrinsic characteristic of certain species. For example, there is an abundance of females in cyclically parthenogenetic species (for example, aphids, cladocera, rotifers), in sequential hermaphrodite species and in many haplodiploid species, such as ants, bees, wasps and mites. The parasite has a skewed likelihood of encountering male versus female hosts in populations with strongly biased sex-ratios and sex-specific adaptations are likely to occur. This study characterizes the host sex-specific effects of parasites in sexually dimorphic hosts with biased sex-ratios to explore this hypothesis.

We use the cyclically parthenogenetic crustacean *Daphnia magna *and its natural bacterial parasite, *Pasteuria ramosa*, as a model system for host-parasite interactions. In this system, infection success depends on the combination of host and parasite genotypes [7]. Host susceptibility correlates with the ability of the parasite to attach to the host's esophagus, a process that does not depend on host sex [8]. Even though females and males are genetically identical (sex is environmentally determined and the sexual dimorphism is due to phenotypic plasticity [9]), there is clear sexual dimorphism [10] and, as is typical for cyclic parthenogenetic animals, natural populations of *D. magna *are strongly female biased. As such, parasites of *D. magna *will typically encounter females considerably more often than males and might be adapted to exploit that particular host type.

Most published studies of host-parasite interactions using *Daphnia *focus exclusively on female hosts (for an exception see [11]). Those studies include detailed descriptions of parasite-driven changes to their female hosts, such as the *P. ramosa*-induced castration of *D. magna*. When proliferating within females, *P. ramosa *induces the reallocation of resources usually spent in egg production to the production of somatic tissue. This results in female gigantism and, consequently, in increased carrying capacity for parasite proliferation inside infected females. This link between parasite fitness and host gigantism is observed for many species [12-14] and has specifically been seen in our *P. ramosa*-*Daphnia *model [15,16]. However, *P. ramosa *is rarely exposed to this "host type" in natural populations and whether infection of males with *P. ramosa *results in castration, gigantism and subsequent increase parasitic capacitance is unknown.

The first aim of this study was to investigate if the two host sexes represented different environments for the parasite. For several genetically distinct host clones, we recorded the differences between host sexes of the same host clone (sexes are genetically identical) in parasite infectivity, virulence, proliferation and fitness. The second aim of our study was to test if parasite-induced castration and gigantism, described for infected female *D. magna*, are also observed in infected males. If these symptoms are not seen in males, it is consistent with our hypothesis that the parasite adapted to exploit female hosts.

## Results

In a series of experiments (summary in Table 1) using the crustacean host *D. magna *and *P. ramosa*, its bacterial parasite, we investigated if parasites exposed to male versus female host individuals differ in the likelihood of successful infection (Experiments 1 and 2), in parasite fitness, in the rate of proliferation within the host (Experiments 2 and 3), and in induced disease symptoms (Experiments 2, 4 and 5).

**Table 1:** Overview of all experiments

No. of the experiment	Experiment	Age at exposure (days)	Exposure duration (days)	Parameters measured	Results figure
1	Likelihood of infection with sex dimorphism during exposure	3	11	- Infection rate	- Figure 1

2	Host gigantism, parasite fitness and virulence	1	2	- Infection rate	
				- Host Survival	- Figure 4
				- Spore counts at death (two clones)	- Figure 3
				- Host body length 21 days post exposure (two clones)	- Figure 5

3	Within host parasite proliferation	1	2	- Spore counts 20 and 27 days post-exposure	- Figure 2a
				- Host body length 20 and 27 days post-exposure (for spore density)	- Figure 2b

4	Host gigantism	3	11	- Host body length 21 days post exposure	- Figure 5

5	Male castration	1	2	- Spermatozoa counts 13 to 26 days post exposure	- Figure 6

### Infection rate and parasite proliferation in male versus female host individuals

**Figure 1:**
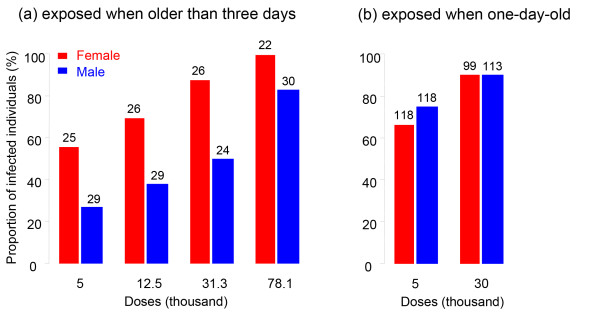
**Proportion of infected male versus female *D. magna *hosts after exposure to *P. ramosa *spores**. **(a) **The data correspond to Experiment 1, with long exposure time (for 11 days) of sexually dimorphic hosts (three-day-old). The proportion of infected increased with spore dose in both sexes (factor "Dose", df = 3, deviance = 34.9, *P *< 0.00001) and was always higher in females than in males (factor "Sex", df = 1, deviance = 27.4, *P *< 0.00001; interaction "Sex × Dose", df = 1, deviance = 1.35, *P *= 0.45). **(b) **The data correspond to Experiment 2, with short-exposure time (for two days) hosts to young to show sexual dimorphism (one-day-old). The infection rate increased with spore dose in both sexes (factor "Dose", df = 1, deviance = 26.5, *P *< 0.00001; interaction "Sex × Dose", df = 1, deviance = 1.33, *P *= 0.24). We did not observe a difference in the proportion of infected females versus males (factor "Sex", df = 1, deviance = 1.6, *P *= 0.21). We pooled the three host clones used in this experiment as they were not significantly different (factor "Host", df = 6, deviance = 8.1, *P *= 0.23). Numbers of replicates are given on top of each bar.

*P. ramosa *had higher infection rates in females when three-day-old individuals were exposed to parasite spores for 11 days (Experiment 1, Linear mixed model, factor "Sex", df = 1, deviance = 27.4, *P *< 0.00001, Figure 1A). The infection rate increased with the dose of parasite spores (factor "Dose", df = 3, deviance = 34.9, *P *< 0.00001, Figure 1A) but the sex difference did not vary with dose (interaction "Sex × Dose", df = 1, deviance = 1.35, *P *= 0.45, Figure 1A). However, when we exposed one-day-old hosts, before the sexual dimorphism becomes apparent, for a short period (48 hours; Experiment 2), we did not observe a difference in the proportion of infected females versus males (Linear mixed model, factor "Sex", df = 1, deviance = 1.6, *P *= 0.21, Figure 1B).

**Figure 2:**
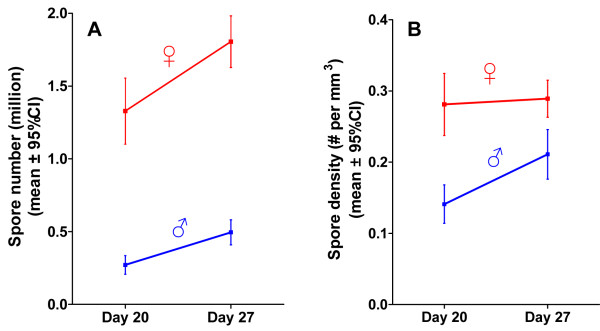
**Number and density of parasite spores in male and female hosts, 20 and 27 days post-exposure**. The *P. ramosa *spore number (**A**) and the density (**B**) were higher in females *D. magna *than in males. Error bars represent the 95% confidence intervals.

Spore counts (Figure 2A) and spore densities (Figure 2B) at Day 20 of the experiment were higher in females than in males (Experiment 3; with a short period of exposure of young, sexually immature host individuals). This suggests that the rate of spore production in females was higher than in males in the first 20 days of the experiment. As animals were exposed to the parasite before sex differentiation, the differences in spore counts and densities are unlikely to be caused by differences in the number of spores ingested (that is, differences in the initial inocula). Between Days 20 and 27 of the experiment, the rate of spore production (slope in Figure 2A) no longer differs significantly between the sexes (Two-way ANOVA (log(spore number)): n = 142; factor "Sex" df = 1, F = 289.37, *P *< 0.00001; factor "Day" df = 1, F = 31.96, *P *< 0.00001; interaction "Sex × Day" df = 1, F = 1.62, *P *= 0.2). Parasite density increased in males during the later phase of parasite proliferation (Welch's t-test: df = 61.67, t = -3.23, *P *= 0.002; Figure 2B), but did not in females (Welch's t-test: df = 59, 03, t = -0.29, *P *= 0.77; Figure 2B).

**Figure 3:**
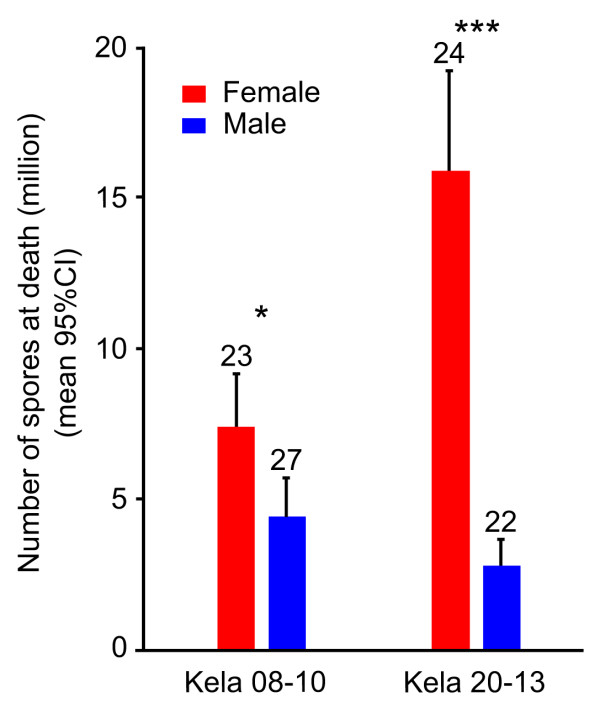
**Number of spores at death in male and female hosts**. *P. ramosa *had higher fitness in females than in males in both *Daphnia *(Experiment 2, Kruskal-Wallis rank test, Clone Kela-08-10: df = 1, χ^2 ^= 6.2, *P *= 0.01; Clone Kela-20-13: df = 1, χ^2 ^= 32.1, *P *< 0.00001). Error bars are 95% confidence intervals.

At parasite induced host death, females harbored many more spores than males (Experiment 2, Clone Kela-08-10: Kruskal-Wallis rank test, df = 1, χ^2 ^= 6.2, *P *= 0.01; Clone Kela-20-13: df = 1, χ^2 ^= 32.1, *P *< 0.00001, Figure 3).

### Effects of parasites on males versus female hosts

Over all the experiments, we found no significant difference in mortality before Day 14 (the earliest it is possible to reliably check for infection status) between male and female hosts, and between host clones (Two-way ANOVA (log(number of dead individuals before Day 14)): "Host clone", df = 10, F = 2.5, *P *= 0.08, "Sex", df = 1, F = 1.41, *P *= 0.26). Individuals dead before Day 14 were excluded from further analysis.

**Figure 4:**
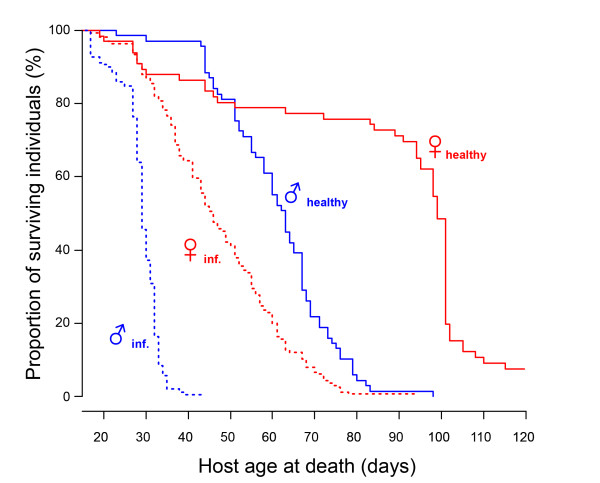
**Survival of control (solid lines) versus infected (dotted lines) of male and female hosts**. Control *D. magna *live about twice longer than infected ones. *P. ramosa *reduces the lifespan of both female and male *Daphnia*. We did not find a statistically significant difference between sexes of such lifespan reduction. The median lifespan was reduced in both sexes by exactly 50%.

We monitored lifespan of infected versus control male and female hosts (Experiment 2) and showed that control *Daphnia *of both sexes lived longer than their infected counterparts (females: Log-rank test: n = 232, df = 1, χ^2 ^= 111, *P *< 0.00001; males: n = 260, χ^2 ^= 190, df = 1, *P *< 0.00001, Figure 4). We did not detect a significant difference in cost of infection on survival between male and female hosts (Coxph: factor "Infection status", Exp(coef) = 15.61, Z = 10.8, *P *< 0.00001, factor "Sex", Exp(coef) = 7.41, Z = 7.67, *P *< 0.00001, factor "Infection status × Sex", Exp(coef) = 1.37, Z = 1.035, *P *= 0.3). The median lifespan was reduced by about 50% in both sexes (Figure 4).

**Figure 5:**
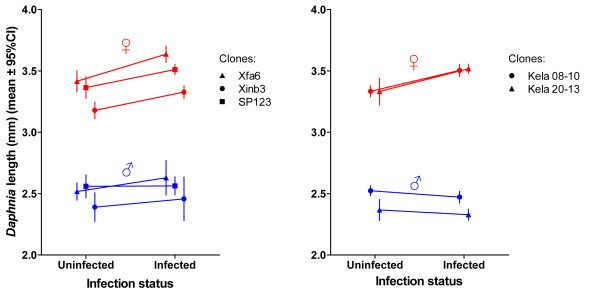
**Body length of infected versus uninfected male and female hosts, 21 days post-exposure**. *P. ramosa *induced gigantism in female *D. magna *but not in males (see Table 2 for details). Error bars show 95% confidence intervals. Stars in the legend represent significant differences based on t-test comparisons (*P *< 0.0001). The results for females remain significant (*P *< 0.01) when corrected for multiple testing. Note that *P *> 0.2 in all comparisons for males.

We tested whether *P. ramosa *induces gigantism in its hosts in two experiments (Experiments 2 and 4). We found that infected females were larger than uninfected females (Figure 5, Table 2), while body size of infected males was not significantly different from uninfected males (Figure 5, Table 2).

**Table 2:** Summary of differences in body length between infected and uninfected female and male hosts

	*D. magna *clone	Sex	N	t-ratio	*P*-value
	Kela 08-10	Female	42	5.24	**< 0.0001**
Exp.		Male	38	-1.62	0.95
	
No. 2	Kela 20-13	Female	45	3.71	**< 0.0001**
		Male	34	1.34*	0.21

	Xfa6	Female	27	4.53	**< 0.0001**
		Male	27	0.95	0.35
	
Exp.	Xinb3	Female	27	4.73	**< 0.0001**
No. 4		Male	27	0.7	0.49
	
	SP-1-2-3	Female	27	3.74	**< 0.0001**
		Male	37	0.08	0.53

Based on t-test comparisons, the parasite-induced gigantism only in female hosts. The results for females remain significant (*P *< 0.01) when corrected for multiple testing. The means of body length are represented in Figure 5. * Result obtained with a "Welch's t-test" to control for unequal variances.

**Figure 6:**
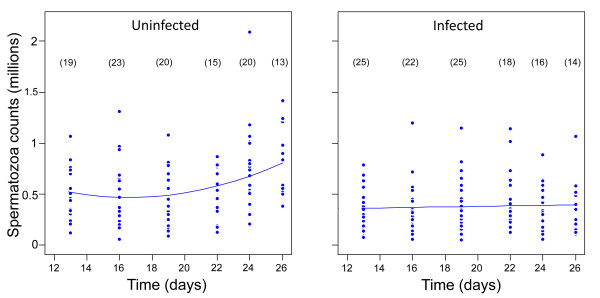
**Spermatozoa counts in uninfected (left) and infected (right) male hosts**. The counting was performed over the period where most of adult infected male *Daphnia *are expected to have spermatozoa and have survived infection (based on Figure 4). Infected males have fewer spermatozoa than uninfected males (linear regression controlling for variance due to the "Sampling day"; "Infection status", df = 1, F = 25.2, *P *< 0.001). While spermatozoa counts increased in uninfected males (linear regression with quadratic term, ("Age")*^2*, df = 1, F = 10.35, *P *= 0.001 and "Age" df = 1, F = 3.39, *P *= 0.07, left panel) it did not in infected males (linear regression, "Age", df = 1, F = 0.05, *P *= 0.82, right panel). Time is given as days after exposure to the parasite. Sample sizes are given in brackets.

As seen previously during infection with *P. ramosa*, infected female hosts in our experiments did not produce eggs. We tested whether infected males showed signs of castration (Experiment 5) by looking for and counting spermatozoa. All adult males had spermatozoa, but infected males had significantly lower counts (linear regression controlling for variance due to the factor "Age"; factor "Infection status", df = 1, F = 25.2, *P *< 0.001, Figure 6). Spermatozoa counts increased with age for uninfected individuals (linear regression with quadratic term, factor ("Age")*^2*, df = 1, F = 10.35, *P *= 0.001 and factor "Age" df = 1, F = 3.39, *P *= 0.07, Figure 6, left panel), but not for infected individuals (linear regression, factor "Age", df = 1, F = 0.05, *P *= 0.82, Figure 6, right panel).

## Discussion

We used a host whose populations are typically strongly female-biased and investigated infection-associated characteristics to look for sex-specific differences. Our results show that male and female *Daphnia *differ in the likelihood of becoming infected upon exposure to *P. ramosa *parasite spores, but only when they already show a sexual size dimorphism, about three days after birth (Figure 1). We also showed that parasites infecting females proliferate at a higher rate, reach a higher density (Figures 2 and 3) and have a higher fitness (measured as total spore count at host death). However, longevity is unaffected by host sex (Figure 4). Finally, we showed that both sexes suffer from a reduction in fecundity (sperm and egg counts) when infected (Figure 6), but only females experience an associated increase in body size (gigantism) (Figure 5). Together, these data are consistent with the hypothesis that this parasite is adapted to specifically exploit the considerably more common female hosts.

### Male and female hosts represent different environments for parasites

As in many organisms, differences in morphology and physiology between male and female *Daphnia *are minimal in very young individuals (for example, within the first three days of life there is no size difference) with sexual size dimorphism developing from the third instar onwards. The developmental timing of the size sexual dimorphism may explain why females were infected at a higher rate than male hosts when encountering the parasite at adult stage, while infection rates were equal for exposures at an early juvenile stage. *Daphnia *passively capture *P. ramosa *spores from the water by filter feeding, and larger animals have higher filtration rates. Thus, as females grow faster and to a larger size, they may take up more spores with their food than males. A bias in parasite exposure due to sex size dimorphism has been proposed to explain part of the sex bias in infection rates in other animals [17] and between species of *Daphnia *[18,19]. However, further investigation on the relative roles of body size or sex in isolation on the likelihood of infection remains to be done. A sex difference in likelihood of infection means that parasites will experience the female host environment more often, even if the sex ratios were not biased.

Infected female *D. magna *lived about 1.5 times longer (median) than infected males, but the cost of the parasite on host lifespan was approximately the same in both host sexes (Figure 4; see differences between infected versus uninfected individuals of the same sex). The longer lifespan of female hosts means that parasites infecting females have the opportunity for more cell divisions within the host and, consequently, more opportunity to adapt to this host's characteristics [20,21]. The biased sex-ratio, the higher infection rate and the higher number of parasite cell divisions within female hosts in this system suggest that selection on *P. ramosa *is stronger on traits favoring the exploitation of female host characteristics than on those favoring the exploitation of male characteristics (anatomic and/or physiologic). While these results are not direct evidence for adaptation, the observed conditions theoretically favor parasite adaptation to the female host environment [6].

The higher parasite fitness (estimated as the production of spores, the parasite's transmission stage) recorded in female hosts may be explained by a combination of female *Daphnia*'s longer lifespan (Figures 3 and 4), larger body size (Figure 5) and higher spore production rate in the first 20 days of infection (Figure 2A). Larger body size itself increases the total capacity for spore production in an infected host [15]. This is supported by a gradual increase of parasite density in males (which do not change size when infected) while density in female hosts (which increase in size when infected) stabilizes (Figure 2B). Our result suggests that *D. magna *females are weaker than males in facing *P. ramosa *infection and/or that this parasite is better adapted to exploit female hosts. Since males and females have the same genotype, the difference cannot be due to differences in host genetic composition. The relative contributions of host and parasite life history traits to the higher fitness of *P. ramosa *in female *D. magna *cannot be disentangled. However, the finding discussed below does suggest that this parasite has some traitsthat maximize its fitness, specifically in the female host environment.

### Host sex-specific symptoms

Female *Daphnia *have been repeatedly reported to exhibit parasite-induced gigantism, that is, enhanced body growth, and castration upon infection with *P. ramosa *[for example, [15,22]]. Parasites adapted to manipulate energy budget allocation towards growth have been observed in diverse taxa, including molluscs, crustaceans, vertebrates and plant hosts, and for bacterial, fungal and helminth parasites [23-27]. In the *Daphnia-Pasteuria *system, gigantism has been proposed as a parasite adaptation, and not as a host response, to increase the parasite's lifetime reproductive success, that is, the number of spores produced until host death [15,16]. By inducing host gigantism, *P. ramosa *increases the host carrying capacity, which allows parasites to reach higher spore counts. A higher carrying capacity may also decrease the parasite density temporarily (as confirmed for females in Figure 2B), which may expand host lifespan [15]. This effect, documented for female *D. magna *hosts, had never been investigated for male hosts, the host environment less often experienced by this parasite. Our results show that host body size increases in infected females, but not in males (Figure 5). This suggests that natural selection has favored parasite traits that induce gigantism specifically in *D. magna *females. In females, castration is completed soon after the infection [15]. Males, on the other hand, appear to produce their sperm early in *Daphnia *development [28]. This would mean they already have most of their spermatozoa during initiation of infection, before the parasite gains control over the host. Uninfected male *Daphnia *continue production of spermatozoa at a low rate; infected male *Daphnia*, however, have the number of their spermatozoa plateaued (Figure 6). This suggests that spermatozoa production was stopped or reduced after the parasite gained control over the host. Since castration occurs after most of the resources allocated to sperm production have already been spent, there is likely little benefit for the parasite (for example, no resources to create gigantism).

How the parasite induces host castration and gigantism is still unknown. Previous studies in our and other systems have proposed that gigantism of female hosts is a consequence of castration [12,15,16]. The idea is that preventing investment in reproductive tissues results in more energy allocated to somatic growth. The castration itself may result from a chemical secretion from the parasite that modifies host hormonal regulation, returning mature adult females to an immature hormonal stage [15]. While the castration of female hosts allows re-allocation of significant amounts of resources to somatic tissues, and thus leads to gigantism, that of males has apparently no effect on body size. The same chemical secretion may induce male castration, but due to the small amount of resources reallocated there is no resulting gigantism. There are two potential reasons for minimal resource allocation. It is possible that males allocate the resources in sperm production only early in their development, as suggested by the relatively slow increase of sperm cell counts during adulthood, or males simply have reduced resource investment in spermatozoa production relative to a female's investment in egg production. While female fitness depends largely on the quality and the quantity of eggs, male fitness depends on a trade-off between expenditure on ejaculate and expenditure on obtaining matings ([29], p. 7 in [30]). Male *D. magna *are more likely to invest more in obtaining mates because each mating fertilizes a maximum of two eggs. The relevant resource allocation trade-off in males may be between body size and mate searching activity, and a parasite capable of decreasing male activity might be able to induce male growth. We propose that the parasite ability to induce host castration is an adaptation selected to exploit female hosts and it does not lead to an increased capacity for parasite spore production in males.

## Conclusions

In the same way that parasites are expected to be better adapted to the most common host types [5], we propose that parasites can adapt to the characteristics of the host sex they encounter more often. This is expected to be the case when hosts are sexually dimorphic and represent distinct environments for their parasites. We have recently proposed that host sex differences might be important for parasite evolution and can lead to parasite populations specifically adapting to the characteristics of the common host sex [6]. We discussed different scenarios where such sex specific adaptations can occur [6], including the case of sexually dimorphic host populations with strongly biased sex-ratios.

It is yet to be explored how parasite adaptation to host sex depends on the degree of sex ratio bias and of sexual dimorphism in the host population. Here, using a parasite evolving naturally in strongly female-biased host populations, we show a key difference in the symptoms induced by *P. ramosa *parasites in female versus male *D. magna *hosts, and proposed that this reveals a parasite trait selected for its effect in females. To our knowledge, this would be the first documented example of a parasite trait evolved as a specific adaptation to the more common host sex in nature. Revisiting other cases of parasite-induced host symptoms in light of our findings might identify more examples of parasite adaptations specific to one host sex. For instance, we suggest investigating if other examples of parasite-induced host gigantism are also limited to one host sex and if this occurs in host populations with biased sex-ratios.

## Methods

### Biological material

We used 10 different genotypes (clones) of *D. magna *isolated from different pools in a metapopulation in south-western Finland, where *P. ramosa *occurs naturally [31]. *D. magna *clones from this region are known to produce relatively large numbers of males (laboratory and field observations) but are still strongly female-biased. This allowed us to have male and female host individuals from mothers raised in the same laboratory conditions. Host clones were kept in the laboratory in standardized medium (ADaM [32]) at 20°C, and fed daily with chemostat cultured unicellular green algae *Scenedesmus obliquus*. Per day and per individual host, we provided 2.5 million algae cells for the first three days, 3 million for the next four days, and 5 million afterwards. During the experiments, individual *Daphnia *were kept in 100-mL jars with 80 mL ADaM medium, which was changed weekly. The male-specific long antennules that are vestigial in females allowed us to sex *D. magna *individuals shortly after birth (one-day-old host individuals, as used in some experiments), before the differentiation of major sexual dimorphic traits. Other experiments used *D. magna *individuals that were three-days old, an age at which sexual dimorphism (also in body size) starts to be obvious. When applicable, body length of adult *Daphnia *individuals was measured as the distance from the top of the head to the base of the apical spine under a dissecting microscope.

For the bacterial parasite *P. ramosa*, we used clone C19, which was originally sampled from infected *D. magna *females in a population in Gaarzerfeld, Germany [7]. This parasite genotype is not qualitatively different from other known genotypes in terms of induced host symptoms. Parasite spore suspensions were obtained by homogenizing infected *D. magna *in 500 μL of water. Spores were then counted under phase contrast microscopy (Leica microsystems DM 2500, magnification 400×) with a hemocytometer (Neubauer improved) and diluted to the desired concentration for host exposure (see below). As control, we used placebo suspensions obtained by homogenizing uninfected *Daphnia*. Particular host and parasite clones used in the experiments were not coevolving, which allowed us to specifically test for the factor "sex".

Infections were performed by exposing single host individuals to suspensions of parasite spores. For the larger and sexually dimorphic three-day-old *D. magna *individuals, exposure took place in 100-mL jars filled with ADaM and lasted 11 days (4 days in 20 mL followed by 7 days in 80 mL medium) before individuals were transferred to 80 mL of clean medium. For the smaller, one-day-old individuals, exposure took place in wells of 24-well plates containing 1 mL of ADaM and lasted two days before transfer to jars with fresh medium. The infection status of *D. magna *at the end of the experiments was assessed by checking, with phase-contrast microscopy, single individuals homogenized in 500 μL of medium. Individuals that died before Day 14 of the experiments, largely due to handling during sorting, were excluded because detection of *P. ramosa *infection is less reliable during early stages of infection. An overview of the experiments carried out is given in Table 1.

### Likelihood of infection upon exposure

We tested for a difference in infection likelihood between female and male *Daphnia *hosts in two experiments (Experiments 1 and 2, Table 1). For Experiment 1, we used 30 females and 30 males of *D. magna *clone SP1-2-3 for each of five treatments corresponding to exposure to different doses (on a log-linear scale) of parasite spores: control (placebo obtained as described above), 5,000, 12,500, 31,300 or 78,100 parasite spores per jar. At exposure, hosts were three-days old. Eleven days after exposure, *Daphnia *were transferred to fresh medium and 21 days after that, we inspected all individuals (n = 264, excluding 36 that died before Day 14 of the experiment) for the presence of infection with the naked eye. *P. ramosa *infections produce very clear symptoms visible by eye. By Day 20 post infection 100% of the infected hosts show these symptoms. For Experiment 2, we exposed very young (one-day-old) animals, which do not yet show sex differences in traits, such as body size. We used 20 males and 20 females of each of 7 *D. magna *clones (Kela 08-10, Kela 10-01, Kela 12-06, Kela 18-11, Kela 20-13, Kela 28-08 and Kela 39-01) for exposure to each of 2 doses of parasite spores: 5,000 or 20,000 spores per well. As control, we used 14 control animals per clone and sex exposed to a placebo parasite suspension. Individuals dying during the experiment were recorded daily and stored for later analysis. We stopped the experiment 120 days after exposure (when all infected and most control individuals had died) and checked infection status of every individual (n = 582, excluding 174 that died before Day 14 of the experiment).

### Parasite virulence, fitness and proliferation

To measure the parasite's effect on lifespan of male and female hosts, we used longevity data collected in Experiment 2 (details above and in Table 1). Specifically, the survival analysis was done on lifespan data collected daily for infected (from both dose exposures) and healthy individuals from seven *D. magna *clones. All individuals dead before Day 14 of the experiment were removed from the analysis, and the six control females that were still alive at Day 120 of the experiment were censored. To estimate parasite fitness, we counted the number of spores at death for two of the seven host clones (n = 49 for Kela 08-10 and n = 46 for Kela 20-13 in Experiment 2).

To test for host sex differences in the rate of within-host proliferation, we counted spores in two groups at two different times after exposure (Experiment 3, Table 1). Individual *Daphnia *(clone SP1-2-3) exposed to 20,000 spores when one-day-old were killed, measured and homogenized for counting parasite spores (as described above) at Day 20 (37 females and 29 males) or at Day 27 (40 females and 36 males) of the experiment. We stopped the experiment at Day 27 because approximately 50% of the males were dead after that period (Figure 3). The number of parasite spores was estimated by homogenizing individual hosts in 0.5 mL of medium, and counting a subsample of this suspension using a hemocytometer (Neubauer improved). For each individual, we also calculated the density of spores by dividing the number of spores by the host body volume (body volume = 0.2418 × body length^2.593 ^[33]). Note that because the formula to calculate host body volume was established for females which have a brood pouch, it is possible that male body volume was underestimated and, consequently, that parasite density in male hosts was overestimated. If this was the case, the differences in densities we found would be even higher. For the analysis of parasite proliferation, we used the difference in parasite number and in parasite density between Days 20 and 27.

### Host castration and gigantism

To test for parasite-induced gigantism, we measured body length of 21-day-old live infected and non-infected individuals from Experiment 2 (clones Kela 08-10 and Kela 20-13) and from an extra dedicated experiment (Experiment 4, Table 1). Here, three-day-old males (n = 25) and females (n = 25) from each of three *D. magna *clones (Xinb3, SP1-2-3, XFa6) were exposed to 30,000 *P. ramosa *spores for 11 days. As controls, we used 13 males and 13 females per clone exposed to a placebo suspension. Twenty-one days after exposure, we measured the body length of all individuals still alive (n = 184) and recorded their infection status.

To test for the effect of parasite infection on spermatozoa production in *D. magna *males (Experiment 5, Table 1), one-day-old males (clone SP1-2-3) were exposed individually (n = 30 per group of the same age) in 20 mL of ADaM medium to 100,000 *P. ramosa *spores (expected to result in 100% infection rates) or to a placebo (control) suspension (n = 25 per group of the same age). The number of spermatozoa was estimated by homogenizing individuals in 50 μL of medium, and counting a subsample of this suspension using a hemocytometer (Neubauer improved). We estimated the number of spermatozoa in control and infected males at ages 13 (is the approximate age for sexual maturity), 16, 19, 22, 24 and 26 days. Individual males were exposed to 50 μL of 2.5% nicotine (15 minutes in the dark), which stimulates muscle contractions and results in the release of mature spermatozoa. Spermatozoa counts were performed in a total of 120 infected and 110 uninfected hosts (see details in Figure 5).

### Statistical analysis

All analyses were performed with R [34]. To compare the proportion of *P. ramosa *infected individuals between host sexes, we used a generalized linear model (GLM) with a binomial error distribution, and logit link (Experiment 1, n = 211, one host clone, one parasite clone; and Experiment 2, n = 448, seven host clones, one parasite clone; see Table 1). Assumptions on the error distribution were checked by estimating dispersion parameters in GLM; no significant over-dispersion was detected. To study the impact of *Pasteuria *on female and male *Daphnia *survival (Experiment 2 in Table 1), we chose to use the non-parametric log-rank test for its robustness (package "Survival" R [34]). The impact of the parasite on host lifespan was assessed by the interaction between the factors "Infection status" and "Sex" in a Cox proportional hazards model. To test for the difference of parasite spore production in male and female hosts, we used non-parametric tests for their robustness (Experiments 2 and 4 in Table 1). For the other tests (specified in the results), we considered parametric assumptions, checked normality and homoscedasticity of residuals, and transformed data when appropriate (the specific data transformation in each case is reported on when the corresponding results are presented). When comparing the body size of hosts infected versus uninfected, we pooled exposed but uninfected and non-exposed individuals as they did not differ in size (linear model with data from Experiment 2, *P *> 0.5; and with data from Experiment 4, *P *> 0.05).

## Abbreviations

ADaM: Artificial *Daphnia *medium; ANOVA: Analysis of variance; GLM: generalized linear model.

## Competing interests

The authors declare that they have no competing interests.

## Authors' contributions

DD, DE and PL designed the study. DE provided the materials, while DD, PL and LD performed the experiments. DD analyzed the data. DD and DE wrote the manuscript. All authors have read and approved the manuscript for publication.
